# Schiff-Base-Modified Chitosan-Metal Complexes as Catalysts in CO_2_ Cycloaddition Reactions

**DOI:** 10.1021/acsomega.6c01736

**Published:** 2026-06-30

**Authors:** Jackelinne Camargo de Lima, Rafael Turra Alarcon, Gilbert Bannach, Ana Paula Garcia Ferreira, Carla Cristina Schmitt, Éder Tadeu Gomes Cavalheiro

**Affiliations:** † Instituto de Química de São Carlos, Universidade de São Paulo, Av. Trabalhador São Carlense, 400, 13566-590 São Carlos, SP, Brazil; ‡ Faculdade de Ciências, Universidade Estadual Paulista Campus Bauru, Av. Eng. Luiz Edmundo Carrijo Coube, 14-01, 17033-360 Bauru, SP, Brazil

## Abstract

Chitosan is a renewable and biodegradable polymer with tunable physicochemical properties, making it an attractive platform for catalytic CO_2_ cycloaddition reactions. This study aimed to develop sustainable chitosan-derived Schiff base metal complexes and evaluate their catalytic performance in the cycloaddition of CO_2_ to epoxides under mild conditions. Low-molecular-weight chitosan (LMWC) was obtained by chitosan depolymerization and subsequently functionalized with salicylaldehyde to form a Schiff base ligand through a straightforward synthetic route. The modified material was then coordinated with Ni­(II), Pt­(II), and Cu­(II) ions to generate metal-based catalytic systems. The ligand and its metal complexes were characterized by complementary spectroscopic, structural, and microscopic techniques, which confirmed successful functionalization and coordination, as well as improved thermal stability compared to the parent LMWC. Catalytic activity was evaluated in the cycloaddition of CO_2_ to styrene oxide under mild conditions (80 °C, 1 bar CO_2_, 24 h). The LMWC-derived catalysts exhibited significantly higher conversions (65–82%) than the noncatalyzed reaction (33%), with the Pt­(II) and Ni­(II) complexes showing the best performance. These findings demonstrate the potential of easily prepared chitosan-derived Schiff base metal complexes as sustainable and efficient catalytic platforms for CO_2_ valorization.

## Graphical abstract

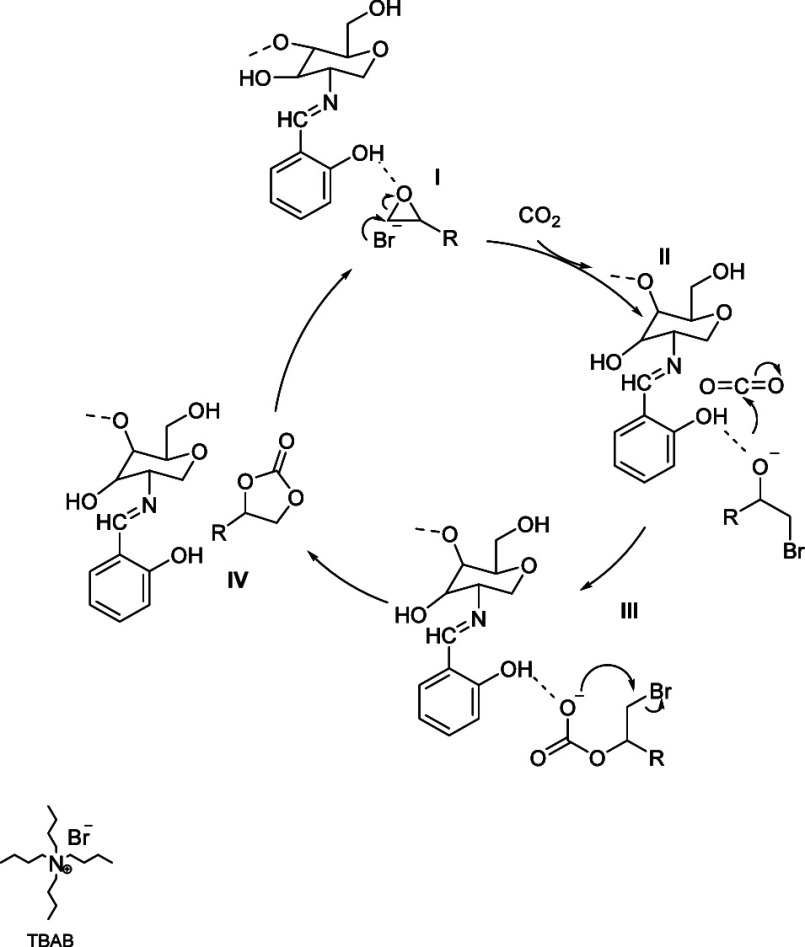

## 1 Introduction

Despite the release of large quantities of carbon dioxide into the atmosphere during combustion, fossil fuels have been the primary energy source in recent decades, enabling substantial increases in human activities. This, in turn, leads to additional CO_2_ emissions, thereby contributing to global warming and severe climate impacts.^1,2^

Carbon dioxide is a recyclable feedstock that plays an important role in organic synthesis and the production of commercially important chemicals. As a potentially important carbon source, the chemical fixation and use of CO_2_ not only help alleviate the “greenhouse effect” caused by excessive CO_2_ emissions but also reduce human dependence on nonrenewable fossil resources, such as coal, oil, and natural gas.^3,4^

Cyclic carbonates are a class of compounds synthesized from CO_2_ and epoxides. Due to their high dipole moment, boiling point, dielectric constant, nontoxicity, biocompatibility, and high structural controllability, these compounds are widely used as electrolyte components in lithium batteries and show promise in the chemical, pharmaceutical, and biomedical industries. Furthermore, cyclic carbonates are often used to synthesize polyurethane monomers, polycarbonates, and polyglycerols.^5^

The literature indicates that using an appropriate catalyst increases the likelihood of the addition of CO_2_ to an epoxide for the synthesis of cyclic carbonates. The most widely used catalysts are homogeneous, such as quaternary ammonium salts, ionic liquids, and metallic complexes.^6,7^ Examples of biobased catalysts for such reactions include pectin derivatives,^8,9^ chitosan,^10,11^ and cellulose.^12^

There has been increasing interest in heterogeneous biopolymeric catalysts. Chitosan stands out among natural polymers due to its low toxicity, low solubility in organic solvents, biodegradability, and biocompatibility. These properties enable the use of chitosan in the agricultural, cosmetic, dental, medical, food, and pharmaceutical industries, as well as for catalysis.^13–16^

Chitosan is a linear polysaccharide obtained from the alkaline deacetylation of chitin by hydrolysis of the acetamide group. The random copolymer is composed of *N*-acetyl-d-glucosamine (GlcNAc) and d-glucosamine (GlcN) units linked by a β (1→4) glycosidic bond. Figure 1 represents the composition of acetylated and deacetylated groups.^17^

**Figure 1:**
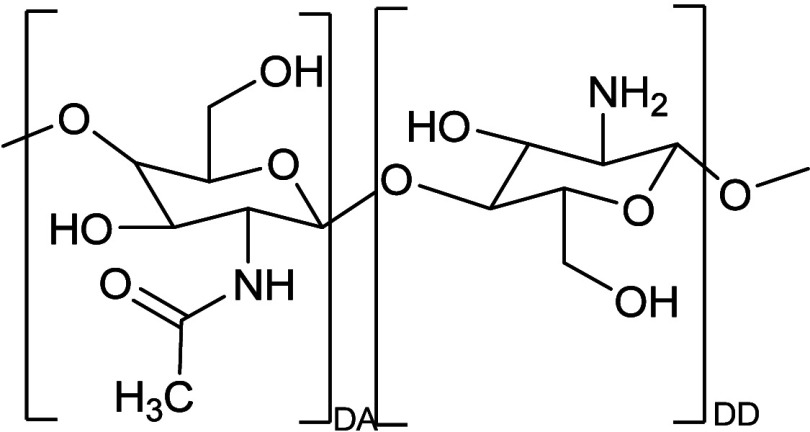
Representation of the structural formula of chitosan. DA = degree of acetylation; DG = degree of deacetylation, when DA ≥ 50% corresponds to chitin and when DA< 50% correspond to chitosan.

Low-molecular-weight chitosan can be prepared by nitrite oxidation, yielding smaller molecules that are more reactive than chitosan itself because of their smaller size and greater mobility.^18^

As in the original structure, LMWC contains functional groups, including primary and secondary hydroxyl groups at carbons C3 and C6, respectively, in addition to an amino group. These sites undergo chemical modification, thereby enhancing the physicochemical properties and biological activity.

In one study, a zeolite-chitosan composite exhibited a greater CO_2_ adsorption capacity than pure zeolite and chitosan and exhibited significant catalytic activity in the chemical fixation of CO_2_ into cyclic carbonates.^11^ Another study used chitosan as a green support to produce a versatile heterogeneous catalytic system. Chitosan was first functionalized with an indigo carmine (IC). Next, metal nanoparticles (i.e., Cu_2_O and CuO) were immobilized on IC-functionalized chitosan (IC–CS). The catalytic activities of Cu_2_O/IC–CS and CuO/IC–CS were investigated for CO_2_ fixation. The results revealed that the use of these biocatalysts for the fixation of CO_2_ with water as a green solvent resulted in a high efficiency and selectivity for the desired products in the shortest time.^19^

Catalysts commonly used in the copolymerization of CO_2_ with epoxides are homogeneous, heterogeneous, and supported and are primarily organometallic compounds. Chitosan and its derivative Schiff bases and metal Schiff base complexes have been used for this purpose.^16^

Schiff bases are synthesized by the condensation of carbonyl groups in aldehydes or ketones with primary amines, forming imine groups. The alteration of peripheral substituent groups in the structures of these compounds gives rise to numerous other materials.

Our research group has investigated the reaction of salicylaldehyde and its 5-bromo, 5-chloro, 5-nitro, 5-methyl, and 5-methoxy derivatives with low-molecular-weight chitosan.^20–27^ This transformation results in a biopolymeric Schiff base anchored on chitosan in −NH_2_-free groups. Such studies include the characterization of the resulting Schiff base and the investigation of the degree of substitution through ^1^H NMR as well as the preparation and characterization of the respective metallic complexes.

The purpose of the present work is to prepare and characterize a biopolymeric Schiff base and some of its metallic complexes and evaluate its application as a catalyst in the CO_2_ fixation reaction.

Thus, in this study, we hypothesized that LMWC, Schiff bases derived from salicylaldehyde, and some of the respective metal complexes can be used as catalysts in CO_2_ fixation reactions. Thus, a Schiff base derived from salicylaldehyde anchored to a chitosan oligomer and its complexes with Ni­(II), Cu­(II), and Pt­(II) were prepared and characterized using spectroscopy (FTIR, ^1^H and ^13^C NMR, and X-ray diffraction), thermal analysis (TGA, DTG, and DTA), and microscopy (SEM). These compounds were subsequently employed as catalysts for the carbonation of styrene oxide, a model reaction for assessing the CO_2_ fixation efficiency, yielding promising results under mild conditions. The novelty here is that, to the best of our knowledge, this is the first attempt to use such LMWCs and respective derivatives in this type of application.

## 2 Materials and Methods

### 2.1 Reagents and Solutions

The reagents used in this study included technical-grade low-molecular-weight chitosan (62 kDa) with a degree of deacetylation (DD) of 80% and a Brookfield viscosity of 20 cps, tetrabutylammonium bromide (≥99%), salicylaldehyde (98% purity), styrene oxide (SO, 97%), and analytical-grade (P.A.) nickel­(II) chloride, all purchased from Sigma-Aldrich (Germany). Glacial acetic acid, NH_4_OH, NaNO_2_, triethylamine, and sodium acetate trihydrate (all P.A. grade, Synth, Brazil), as well as copper­(II) sulfate (P.A. grade, Vetec, Brazil), were also used. Except for chitosan, which was purified as described below, all reagents were used as received. H_2_PtCl_2_ was synthesized according to the procedure described by Barbosa et al.^26^

### 2.2 Purification of Chitosan (Ch)

Chitosan was purified by suspending 5 g of a commercial sample in 1.5 L of a 0.5 mol L^–1^ acetic acid solution under stirring for 24 h. The solution was then filtered, and 50 mL of concentrated NH_4_OH was added until pH 8 was reached. The precipitate was stirred for 1 h and then washed until the pH of the solution was between 7 and 8. The final product was filtered and dried at 40 °C under low pressure.^28^

### 2.3 Preparation of Low-Molecular-Weight Chitosan (LMWC)

For the depolymerization of chitosan, 10 g of the polymer was suspended in 555 mL of a 2% acetic acid solution (v/v) for 24 h. Dissolved oxygen was removed by bubbling N_2_ (g) for 1 h. After cooling the solution to 4 °C, 20 mL of a 0.05 mol L^–1^ NaNO_2_ solution was added, and the temperature was maintained at 4 °C for 24 h. The product was precipitated with NaOH, centrifuged at 10,000 rpm for 10 min, washed until the pH reached 8, and dried at 40 °C.^29^

### 2.4 Synthesis of Low-Molecular-Weight Chitosan Schiff Bases (LMWC_s_)

The chitosan oligomer was dispersed in 300 mL of 0.15 mol L^–1^ acetic acid in a reactor immersed in a thermostatic bath at 25 °C for 24 h. The temperature was raised to 55 °C, and 100 mL of ethanol was added. Next, 4 mmol of salicylaldehyde was dissolved in 20 mL of ethanol and added dropwise to the reaction system. The product was washed, filtered, and dried in a vacuum oven at 40 °C to obtain the final low-molecular-weight chitosan Schiff bases (LMWC_s_).^30^

### 2.5 Synthesis of Nickel, Platinum, and Copper Complexes

The compounds were synthesized via equimolar reactions between LMWCs and the corresponding metal precursor. The mol quantity of LMWC_s_ was calculated using the estimated molar mass as discussed in Section 3.1.1. One mmol of NiCl_2_·2H_2_O, H_2_PtCl_6_, or C_4_H_6_CuO_4_·H_2_O dissolved in water (5 mL) was added to a solution containing 1 mmol of CO and 2.2 mmol of sodium acetate trihydrate in ethanol at 40 °C. The resulting mixtures were refluxed for 12 h, and the solutions were then refrigerated for 24 h. The precipitates were filtered, washed with water and ethanol, and vacuum-dried. The color of the products was light green [Ni^II^(LMWC_s_)], dark green [Cu^II^(LMWC_s_)], and light brown [Pt^II^(LMWC_s_)].

### 2.6 Characterization

#### 2.6.1 Average Viscosimetric Molar Mass

Intrinsic viscosity was determined using a Lovis 2000 M automatic rolling sphere microviscosimeter (Anton Paar, Austria) at 25 °C. Ch and LMWC were solubilized in a buffer solution of 0.30 mol L^–1^ acetic acid and 0.20 mol L^–1^ sodium acetate, pH 4.5. The buffer solution was first analyzed, and five solutions (previously filtered using 45 μm microfilters) were established with different concentrations (0.30, 0.40, 0.50, 0.60, and 0.70 mg L^–1^). The mean runoff time was obtained in triplicate for each solution. The average viscosimetric molar masses of the polymer were calculated from the Mark–Houwink–Sakurada equation:

$$[\eta ]=K_{m}{M_{v}}^{\alpha }$$ 1

in which [η] is the intrinsic viscosity of the solution, *K*_m_ and α (0.076 and 0.796, respectively) are polymer constants that depend on temperature and solvent (characteristic constants of the geometry and flexibility of the molecule that vary according to the degree of acetylation of chitosan), and *M*_v_ is the viscosimetric molar mass of the oligomer.^17,31^

#### 2.6.2 Fourier Transform Infrared Spectroscopy (FTIR)

Infrared spectra of the chitosan oligomer and Schiff bases were recorded in an FTIR IRAffinity1 spectrophotometer (Shimadzu, Japan) in the range from 4000 to 400 cm^–1^, with a resolution of 4 cm^–1^. The spectra were obtained using potassium bromide (KBr) pellets prepared with solid samples previously dried in an oven at 40 °C. Samples (5.0 mg) were mixed with 100 mg of dried KBr. This mixture was homogenized in an agate mortar and formed into a pellet using a hydraulic press.

#### 2.6.3 ^1^H NMR

^1^H NMR spectra were obtained at 70 °C in a Premium Shielded spectrometer (model 500/54, Agilent Technologies, USA) operating at 500 MHz. The sample (10.0 mg) was dissolved in 1.0 mL of D_2_O with 1% HCl (v/v), and the solution was stirred for 24 h.

The solid-state ^13^C NMR spectra were acquired on a Bruker Avance III-400–9,4 T spectrometer (399.94 MHz for 1H, Bruker BioSpin, Germany), equipped with a 4 mm magic-angle-spinning (MAS) probe, operating at 5 kHz.

#### 2.6.4 Thermal Analysis

Thermogravimetric analysis (TGA), derivative thermogravimetric (DTG) analysis, and differential thermal analysis (DTA) were performed, and curves were acquired in a simultaneous TGA/DTA SDT-Q600 modulus controlled by the Thermal Advantage Software v.2.5.0.256 (both from TA Instruments, USA). TGA/DTG/DTA curves for all complexes were obtained under a dry-air atmosphere at a flow rate of 50 mL min^–1^ in an open α-alumina sample holder, using a sample mass of approximately 6.0 ± 0.1 mg and a heating rate of 10 °C min^–1^ from 25 to 1000 °C. The TGA modulus was calibrated for mass and temperature using Zn^0^, according to the manufacturer’s instructions.

#### 2.6.5 X-ray Diffraction

X-ray diffraction was performed using a Bruker X-ray diffractometer, model D8 Advance (Bruker, Germany), equipped with a Cu X-ray source (Kα = 1.5418 A) equipped with a PSD LYNXEYE detector (Bruker AXS GmbH, Germany). Measurements were performed in θ/2θ coupled mode, with continuous scanning from 5 to 60°, a step of 0.02°/step, and an accumulation time of 0.5 s per step.

#### 2.6.6 Scanning Electron Microscopy (SEM)

SEM/EDS analyses were performed using a LEO 440 scanning electron microscope (ZEISS, Germany) coupled to an Oxford 7060 EDS detector and an ISIS System Series 300 microanalysis system (Oxford Instruments, UK) equipped with a SiLi Pentafet ATW II (atmosphere thin window) detector with a 133 eV resolution at 5.9 keV and an active area of 10 mm^2^ (Oxford Instruments, UK).

#### 2.6.7 Carbonation of Styrene Oxide Using CO_2_

Styrene oxide (SO; 1.5 mmol), tetrabutylammonium bromide (TBAB; 4.9 mg), and the chitosan derivative catalyst (15.0 mg) were placed in seven different glass vials with magnetic stirrer bars. The vials were placed into a high-pressure reactor and loaded with 1 bar CO_2_ gas (99.9%). The reactor was placed in an aluminum base-plate jacket and maintained at 80 °C for 24 h. Two blank reactions were performed for comparison: one containing only SO and the second containing SO and TBAB in the absence of the chitosan derivative catalyst. The reaction products were subsequently analyzed by FTIR and ^1^H NMR to determine conversion.

## 3 Results and Discussion

### 3.1 Synthesis and Spectroscopic Characterization

The complexes derived from biopolymeric Schiff bases of salicylaldehyde and chitosan (LMWC_s_) were obtained by a complexation reaction with the metallic precursors NiCl_2_·2H_2_O, H_2_PtCl_6_, and C_4_H_6_CuO_4_·H_2_O, as shown in Figure 2.

**Figure 2:**
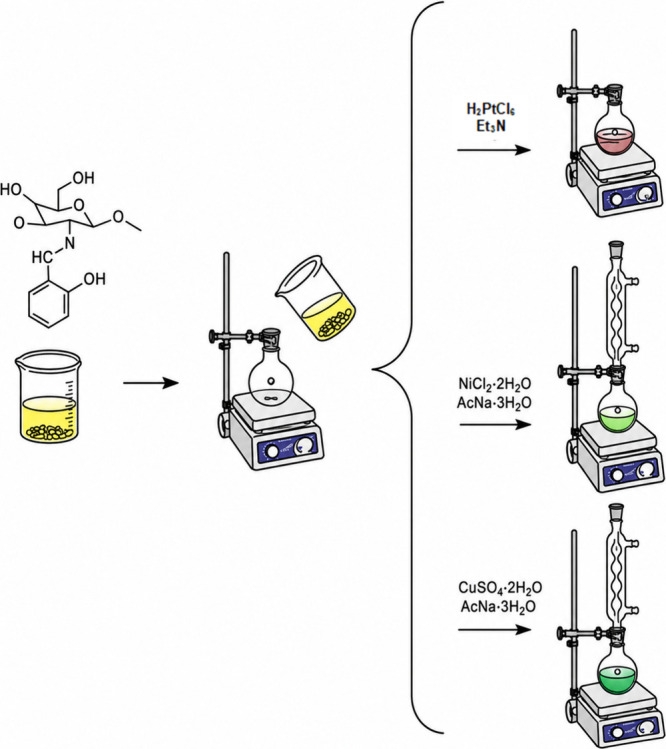
Schematic representation of procedures adopted for the synthesis of complexes under the conditions described in the experimental section.

#### Determination of the Mean Degree of Deacetylation ( $\overset{®}{\mathrm{DD}}$ ) of Chitosan by Hydrogen Nuclear Magnetic Resonance (^1^H NMR)

3.1.1

The mean degree of deacetylation ( $\overset{®}{\mathrm{DD}}$ ) and structural characterization of Ch and LMWC were performed with ^1^H NMR analysis (Figure 3). Using eq 2, it was possible to determine the degree of deacetylation ( $\overset{®}{\mathrm{DD}}$ ) from the ratio between the integration of the area under the peaks in the region from 4.1 to 3.6 ppm ( $A_{H_{3‐6}}$ , $A_{H_{2}}$ ) and the integration of the area referring to the signals of the hydrogen atoms of the methyl group at 2.4 ppm ( $A_{{\mathrm{CH}}_{3}}$ ).

$$\overset{®}{\mathrm{DD}}=1-\frac{A_{{\mathrm{CH}}_{3}}}{A_{H_{2}}-A_{H_{3}‐H_{6}}/6}\times 100$$ 2

**Figure 3:**
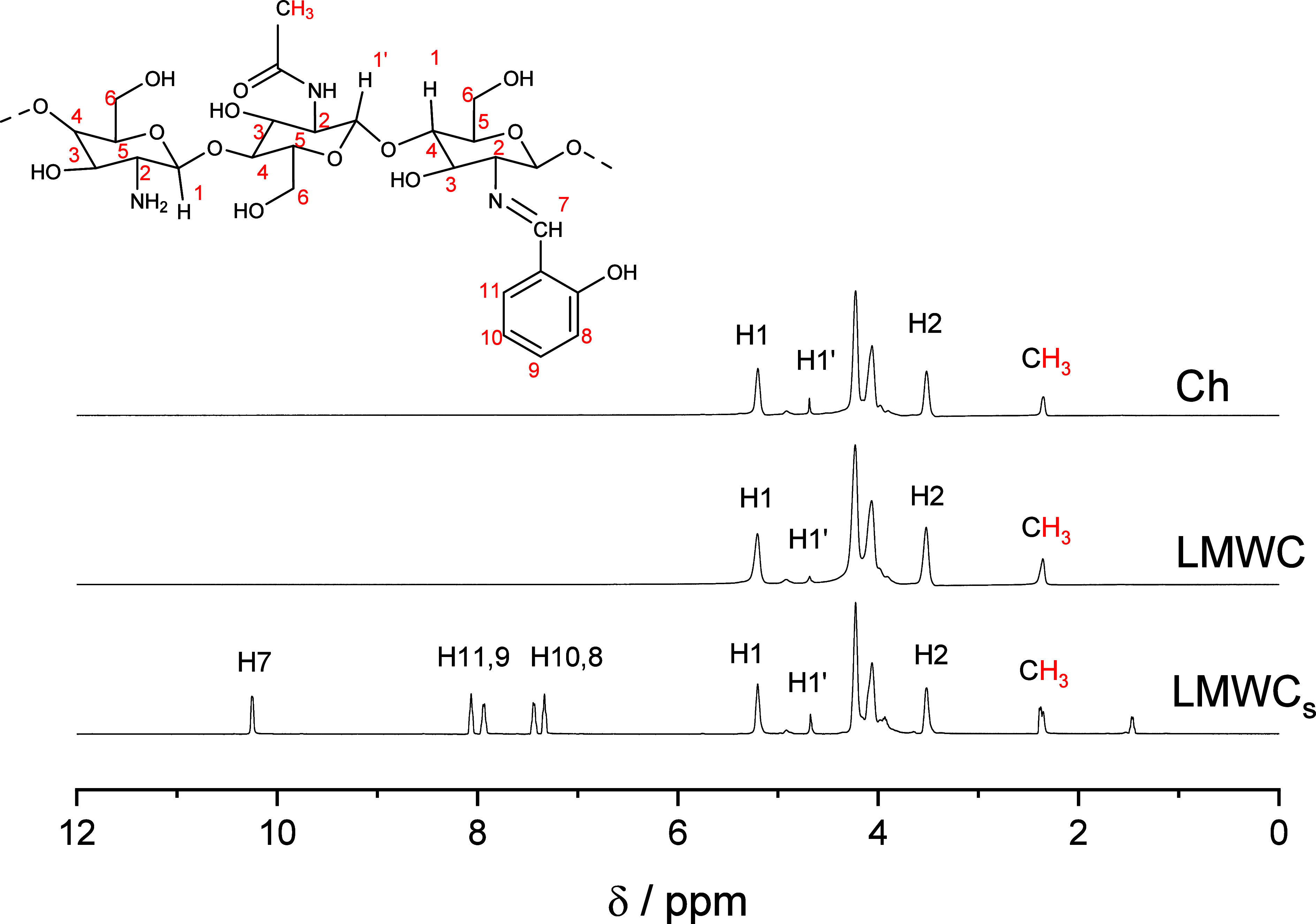
^1^H NMR spectra of Ch, LMWC, and LMWC_s_ (10 mg) in D_2_O/HCl (1%) at 70 °C.

The mean degrees of deacetylation ( $\overset{®}{\mathrm{DD}}$ ) were 82% and 80% for Ch and CO, respectively.

The signal of anomeric hydrogen atoms referring to the deacetylated unit (H_1_) was found at 5.3 ppm, and the hydrogen signal of acetylated monomers corresponded to 5.0 ppm (H_1′_). Signals corresponding to hydrogens linked to carbons 3, 4, 5, and 6 of GlcN units and GlcNAc of the polymer were found in the region between 4.1 and 4.3 ppm. The signal at 3.6 ppm (H_2_) may be attributed to hydrogen bonded to carbon 2 in the glycopyranoside ring of the GlcN units. Hydrogen atoms in the acetamide groups exhibited a signal at 2.4 ppm, which may be attributed to hydrogens of the methyl group (CH_3_).^32^

Figure 3 shows the ^1^H NMR spectrum for LMWC_s_. The signal corresponding to the hydrogen of the salicylaldehyde moiety of the imine exhibited a chemical shift of approximately 10.3 ppm (H_7_). Imines are usually unstable in an acidic medium, and the signal may be attributed to the free aldehyde. However, the FTIR spectra confirmed the formation of the imine in a solid state, and the presence of the aldehyde in the ^1^H NMR spectra confirms the formation of the Schiff base.

The hydrogen bonded to carbon 2 of the glycopyranoside ring signal is seen at 3.5 ppm ( $A_{H_{2}}$ ). These signals were used to determine the degree of substitution ( $\overset{®}{\mathrm{DS}}$ ) with salicylaldehyde using eq 3.

$$\overset{®}{\mathrm{DS}}=\frac{A_{H_{7}}}{A_{H_{2}}}\times 100$$ 3

The degree of substitution of the salicylaldehyde-modified oligomer was 51.8%. The solid-state ^13^C NMR spectra also confirm the formation of the Schiff base on the chitosan biopolymeric base. This is evidenced by the clear presence of carbon signals from the aldehyde (Figure S1, Supporting Information).

These data enabled estimating the mean molar mass ( $\overset{®}{\mathrm{MM}}$ ) using eq 4, which involves the mean degrees of deacetylation ( $\overset{®}{\mathrm{DD}}$ ), acetylation ( $\overset{®}{\mathrm{DA}}=100-\overset{®}{\mathrm{DD}}$ ), and substitution ( $\overset{®}{\mathrm{DS}}$ ) determined from ^1^H NMR and using the theoretical molar masses of each unit:

$$\overset{®}{\mathrm{MM}}=\frac{\mathrm{MMMA}\times \overset{®}{\mathrm{DA}}+\mathrm{MMMD}\times \overset{®}{\mathrm{DD}}+\mathrm{MMMS}\times \overset{®}{\mathrm{DS}})}{100}$$ 4

in which MA is the molar mass of the acetylated monomer, MD is the molar mass of the deacetylated monomer, and MS is the molar mass of the monomer substituted with salicylaldehyde. Table 1 displays the molar masses calculated for chitosan and the derivatives prepared in this study. Molar masses from SEC-MALS-IR and viscosimetry are also presented.

**Table 1:** Molar Masses Calculated by SEC-MALS-IR Analysis and Viscometry as well as $\overset{®}{\mathrm{DD}}$ and $\overset{®}{\mathrm{DS}}$

sample	$\overset{®}{\mathrm{Mw}}$ (kDa)	$\overset{®}{\mathrm{Mn}}$ (kDa)	$\overset{®}{\mathrm{Mz}}$ (kDa)	PDI	DP ( $\frac{\overset{-}{\mathrm{Mw}}}{\overset{-}{\mathrm{MM}}}$ )	$\overset{®}{Mv}$ (kDa)	$\overset{®}{\mathrm{MM}}$ (g mol^–1^)	$\overset{®}{\mathrm{DD}}$ (%)	$\overset{®}{\mathrm{DS}}$ (%)
Ch	135	38	276	3.5	810	36.6	166.6	87.1	
LMWC	62	20	132	3.1	372	6.4	166.4	86.8	
LMWC_s_							221.0	35.0^a^	51.8

a Determined indirectly by the difference between obtained $\overset{®}{\mathrm{DS}}$ values and initial $\overset{®}{\mathrm{DD}}$ values.

The absence of signals referent to the d-2,5-anhydromannose suggests that the purification process was efficient in the removal of such a byproduct of the depolymerization process.^18^

### 3.2 FTIR Spectra

The FTIR spectrum of Ch showed N–H stretching bands characteristic of primary amines at 3500 cm^–1^. However, the O–H band, which is also present in the chitosan structure, likely overlaps with the N–H signal, resulting in a single broad band. Symmetrical and asymmetrical stretching bands for C–H were found at 2925 and 2875 cm^–1^, respectively. The frequency range of the β (1→4) glycosidic linkage appears in the region of 1155 cm^–1^. The bands corresponding to the carbonyl group (C=O) of acetamide substituents are in the 1650 cm^–1^ region.

However, the LMWC_s_ FTIR spectra showed a new band at 1634 cm^–1^, corresponding to the imine bond (C=N), which shows the formation of the Schiff base. Moreover, the band related to C=O stretching between 1660 and 1500 cm^–1^ vanished, hence proving imine formation.

The main evidence of the coordination of metallic ions and the Schiff base group is represented by the displacement of the C=O stretching band from 1634 cm^–1^ in the free ligand to 1627 Ni­(II), 1604 Pt­(II), and 1613 cm^–1^ Cu­(II).

The FTIR spectra for all compounds are shown in the Supporting Information (Figures S2–S4). Table 2 displays the main bands observed in these spectra.

**Table 2:** FTIR Data for the Different LMWC and Derivatives (LMWC, LMWC_s_, and [M^II^(LMWC_s_)], M = Ni, Pt, and Cu)

compounds	*v*(N–H) *v*(O–H)	*v*(C–H)	*v*(C=0)	*v*(C=N)	*v*(C=C)	*v*β(C–O–C)
LMWC	3430	2934/2880	1669			
LMWC_S_	3437			1634	1572	1066
[Ni^II^(LMWC_s_)]	3279			1627	1546	1045
[Pt^II^(LMWC_s_)]	3310			1604		1037
[Cu^II^(LMWC_s_)]	3264			1613	1546	1042

Despite the many coordination modes, a general structure for these complexes was proposed on the basis of the literature (Figure 4).^21^ The metal ions Ni­(II), Pt­(II), and Cu­(II) coordinate with the bidentate ligands through electron pairs in the nitrogen of the imine and oxygen of the aromatic ring, forming square-planar compounds.

**Figure 4:**
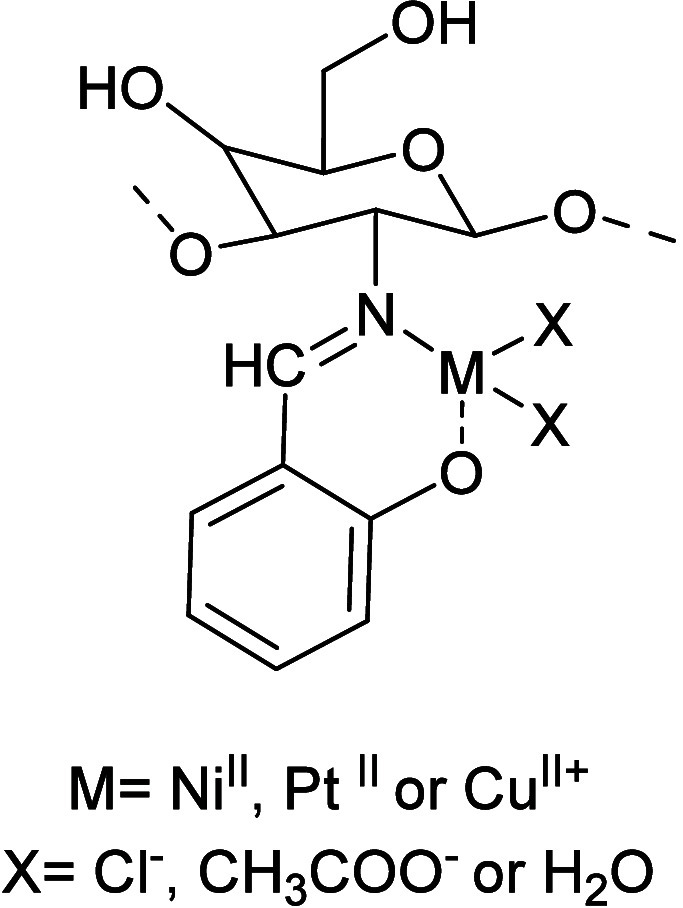
Possible coordination modes for complexes [M^II^(LMWC_s_)] derived from low-molecular-weight Schiff base.

Raman spectra were obtained to characterize the complexation between the Schiff base LMWC and the Ni­(II), Pt­(II), and Cu­(II) ions. However, only the [Cu­(LMWC_s_)] complex (Figure S5, Supporting Information) had the expected bands. Other complexes exhibited fluorescence, which impeded spectral acquisition.

Low-intensity bands were found in the Raman spectrum corresponding to the symmetrical Cu–N stretching vibration at 620 and 651 cm^–1^. Moreover, bands attributed to the asymmetric Cu–O stretching vibration were found at 343 and 462 cm^–1^, suggesting the formation of the metal complex.^33^

Although other analytical techniques could provide additional information regarding the oxidation states and coordination environment of the metal centers, the formation of the complexes was supported by the combined spectroscopic and thermal characterization results obtained in this work, including the characteristic FTIR band shifts after metal coordination and the XRD profiles of the thermal decomposition residues containing metal-derived phases as presented below.^21–27^

### 3.3 Thermal Analysis

The thermal stability and the loss of mass of low-molecular-weight chitosan LMWC and low-molecular-weight chitosan Schiff base were investigated by thermogravimetry. TGA/DTG/DTA curves for all compounds were obtained under atmospheric air. Similarity was found in the events for Ch and the LMWC. Thus, the biopolymer’s structure was preserved despite depolymerization. The TGA curves exhibited three distinct mass loss steps (Figure 5a).

**Figure 5:**
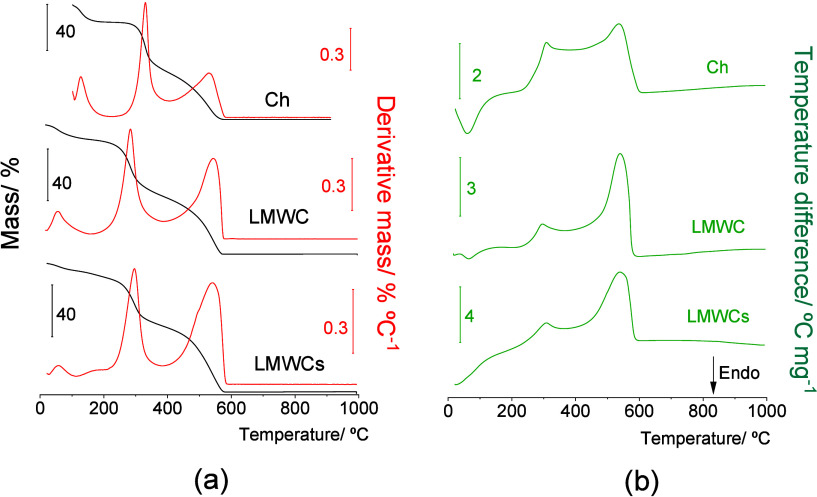
(a) TGA/DTG and (b) DTA curves of chitosan (Ch), low-molecular-weight chitosan (LMWC), and low-molecular-weight chitosan Schiff base (LMWC_S_) under atmospheric air at a flow rate of 50 mL min^–1^ and a heating rate of 10 °C min^–1^; sample mass: 6.0 ± 0.1 mg; α-alumina sample holder.

The mass losses, residue quantities, and temperature intervals for each step of the TG curves for chitosan and its derivatives are listed in Table 3.

**Table 3:** Step Assignments, Temperature Intervals, Mass Loss, and Peak Temperatures Observed in TGA/DTG Curves of Chitosan and Its Derivatives

			TGA/DTG			DTA
compounds	event	**temp. (°C)** ^a^	mass loss (%)	**ratio** ^b^	residue (%)	**peaks (°C)** ^c^
Ch	dehydration	21.2–152.6	15.1	0.9^b^		59.0 (↓)
	1st step	152.6–378.5	43.4			307.6 (↑)
	2nd step	378.5–600.7	41.3			539.2 (↑)
	residue	1000			0.16	
LMWC	dehydration	23.1–151.7	11.1	1.1^b^		60.9 (↓)
	1st step	151.7–374.7	42.7			296.3 (↑)
	2nd step	374.7–597.8	45.4			540.2 (↑)
	residue	1000			0.74	
LMWC_s_	dehydration	23.3–118.6	5.8	1.2^b^		
	1st step	118.6–379.5	42.8			307.4 (↑)
	2nd step	379.5–599.7	51.1			538.7(↑)
	residue	1000			1.46	

a Temperature range.

b Ratio between mass losses in the 2nd step and 1st step, after water loss.

c ↓ = endothermic; ↑ = exothermic.

According to the TG/DTG curves (Figure 5a) for the Ch, LMWC, and LMWC_s_ samples, the first mass loss was related to the release of physically adsorbed water molecules.

The dehydration process was followed by two steps of the polymer degradation. The second and third steps can be attributed to depolymerization and thermal degradation, respectively. In previous work, the evolved gas analysis by TG-FTIR evidenced a complex gaseous mixture mainly composed of ammonia, acetic acid, acetamide, water, monoxide, and carbon dioxide in proportions that are deeply dependent on the DD value, during these two processes.^24,34^

The mass loss ratio between the third and second thermal events indicates greater decomposition in the third event after substitution relative to the starting chitosan. This suggests that the substituted domains are more thermally stable than nonsubstituted domains, decomposing at higher temperatures in the LMWC. Similar results were found for Schiff bases in chitosan.^24^ At the end of the run, the residue values were close to zero.

DTA curves in Figure 5b presented endo and exothermic events that corroborate these observations from TG/DTG curves (Table 3).

For the complexes, the TG/DTG curves (Figure 6a) basically show the same three thermal events for Ch, LMWC, and LMWC_s_. An additional loss of mass was found for [Pt­(LMWC_s_)] at 850 °C, which was attributed to the formation of the respective metallic oxides.

**Figure 6:**
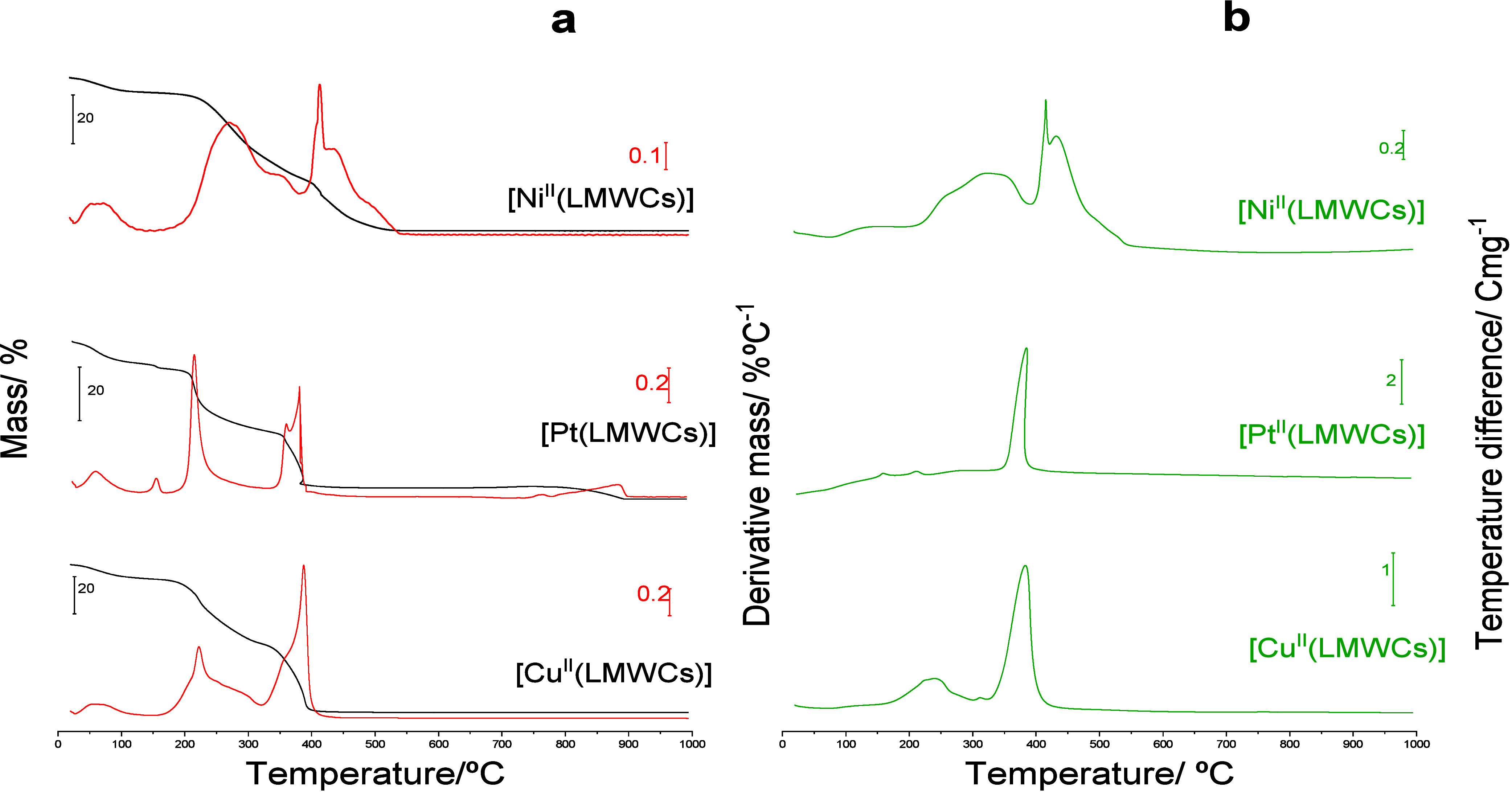
TGA (−)/DTG (−) (a) and DTA (−) (b) curves of complex derivatives of LMWC_s_ under atmospheric air at a flow rate of 50 mL min^–1^ and a heating rate of 10 °C min^–1^; sample mass: 6.0 mg ± 0.1 mg; α-alumina sample holder.

However, differences were observed in the DTG curves at temperatures associated with the third thermal event, indicating the presence of multiple processes. These differences were attributed to the degradation of the complex domains and corresponded to the redox process involving the metal centers.

A noble metal was used; it is possible that atomic species formed in the case of Pt at the end of the third event due to reduction of the ion by the carbonaceous residue from the decomposition, with subsequent oxide formation. This does not occur with Ni and Cu complexes that generate oxides directly. DTA curves (Figure 6b) were not sensitive enough to detect these changes. With the exception of Ni, only one exothermic event was observed in the third step.

The synthesized complexes have different thermal stabilities after dehydration. The platinum complex had the highest thermal stability. Nickel and copper complexes had relatively lower initial thermal decomposition temperatures. The following is the order of thermal stability: [Pt­(LMWC_s_)] (170.5 °C) > [Cu­(LMWC_s_)] (146.6 °C) ≈ [Ni­(LMWC_s_)] (144.1 °C) > LMWC_s_ (118.6 °C).

Comparing the orders of thermal stability observed for the complexes with the order obtained for the free LMWC_s_ ligand, the initial temperature of thermal decomposition of the Schiff base itself was lower (118.6 °C). Therefore, the metals increased the thermal stability. This is an important consideration when using the complexes in catalytic reactions that involve heating steps.

The higher stability of the Pt­(II) complex suggests that it presents a larger interaction with the biopolymeric Schiff when compared with the Cu­(II) and Ni­(II) complexes, probably due to a higher complex stability in this case.

The mass loss percentages at each stage, the temperature ranges, the percentages of residue remaining after burning the material, and the temperatures of the endothermic and exothermic peaks in the DTA curves are summarized in Table 4.

**Table 4:** Data Obtained from TG/DTG and DTA Curves of Biopolymeric Schiff Base Metal Complexes

**TGA/DTG**						DTA
compounds	event	**temp. (°C)** ^a^	mass loss (%)	ratio	residue 1000 °C (%)	peak (°C)
[Ni(LMWC_s_)]	dehydration	18.5–144.1	8.7			74.0 (↓)
	1st step	144.1–379.5	49.6	0.6^b^		324.1 (↑)
	2nd step	379.5–546.7	31.4			415.2/432.7 (↑)
	residue	1000			10.2	
[Pt(LMWC_s_)]	dehydration	21.0–123.8	8.2	1.0^c^		58.1 (↓)
	1st step	123.8–170.5	2.1			211.2 (↑)
	2nd step	170.5–288.3	21.2			386.1 (↑)
	3rd step	288.3–422.0	23.3			
	residue	1000			40.5	
[Cu(LMWC_s_)]	dehydration	20.2–146.6	7.7			
	1st step	146.6–321,5	35.0	1.0^b^		243.2/311.4 (↑)
	2nd step	321.5–496.3	37.0			382.8 (↑)
	residue	1000			20.2	

a Δ*T* = temperature range.

b Ratio between mass losses in third and second steps.

c Ratio between mass losses in fourth and third steps; ↓ = endothermic; ↑ = exothermic.

### 3.4 X-ray Diffraction (XRD) in Characterization of Residues Obtained in Thermal Decomposition of Complexes

The residues from the thermal degradation of the complexes were obtained in a muffle furnace on a preparatory scale under conditions identical to those used in thermogravimetric measurements. Accordingly, heating was carried out to 1000 °C under air at 10 °C min^–1^, with a 3 min isotherm after thermal decomposition. This procedure was applied to all synthesized complexes.

The X-ray diffractograms (Figure 7) were obtained between 5° and 120° (2θ) for the residues of the nickel­(II), platinum­(II), and copper­(II) complexes.

**Figure 7:**
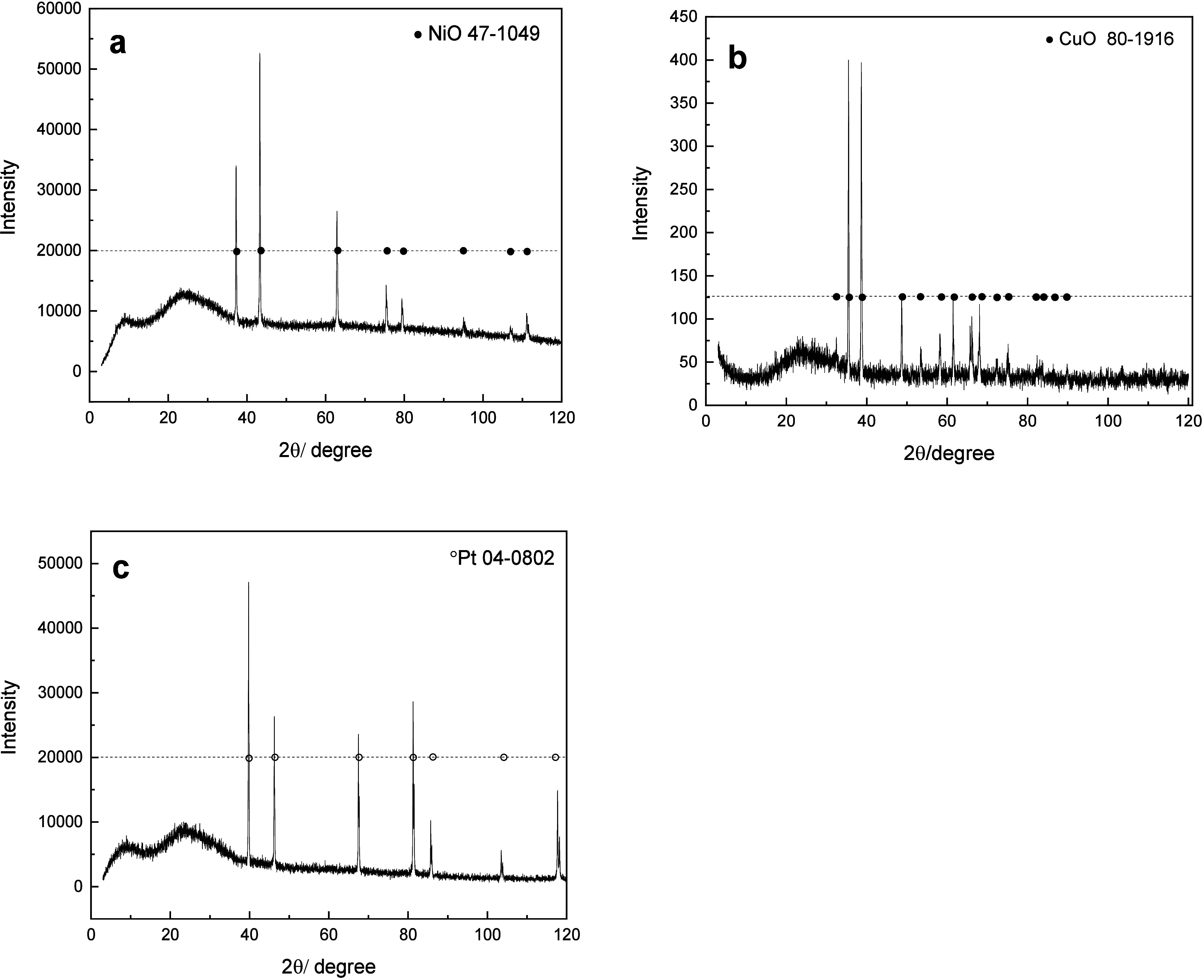
X-ray diffractograms of residues from burning of complexes [Ni­(LMWC_s_)], [Pt­(LMWC_s_)], and [Cu­(LMWC_s_)] (a) NiO, (b) CuO, and (c) Pt. Symbols (filled and open circles) indicate the database values for each product obtained.

Comparing the analysis of the X-ray diffractograms for these residues with the DIFFRAC.EVA database, the residues of the [Ni­(LMWC_s_)] (Figure 7a) and [Cu­(LMWC_s_)] (Figure 7b) complexes have characteristic peaks of the crystalline structure corresponding to the respective oxides: NiO and CuO.

In the diffractogram presented in Figure 7c, the reduced metal Pt^0^ was also obtained as a residue from the burning of the platinum complex. This is usual in thermal analyses, when a large amount of organic matter undergoes decomposition via the carbonaceous material. During the decomposition, this material rich in carbon reduces locally the metallic species, even in an oxidative atmosphere.

### 3.5 Assessment of the Crystallinity Index by X-ray Diffraction (XRD)

Chitosan exhibits a crystalline pattern with characteristic peaks at 2θ = 10° and 2θ = 20°, attributable to intra- and intermolecular hydrogen bonds, respectively.^21^ However, the formation of Schiff bases and coordination with metal ions can lead to a reduction in the quantity of hydrogen bonds due to substitution in the free amino groups of chitosan.^35^

The modification with the salicylaldehyde group exhibited a peak at 2θ = 6°, attributable to new side-chain groups in the biopolymer, which reduced signal intensity, suggesting lower crystallinity.^21^ Coordination with metallic ions also decreased signal intensity and broadened the peaks, suggesting lower crystallinity of the biopolymeric complexes, likely due to a reduction in the number of free groups capable of interacting with one another. Figure 8 shows the diffractograms of chitosan and its derivatives. The crystallinity index was calculated using eq 4.^36^

$$\mathrm{crystallinity}\;\mathrm{index}\;(\%)=\frac{I_{110}-I_{\mathrm{am}}}{I_{110}}\times 100$$ 5

in which *I*_110_ is the maximum intensity at 2θ ∼ 20° and *I*_am_ is the diffraction intensity in the amorphous region at 2θ ∼ 10°. The following was the order of the crystallinity index (%) for the compounds: Ch (65.3) > LMWC (59.8) > LMWC_s_ (26.0) > [Ni­(LMWC_s_)] ≈ [Pt­(LMWC_s_)] ≈ [Cu­(LMWC_s_)].

**Figure 8:**
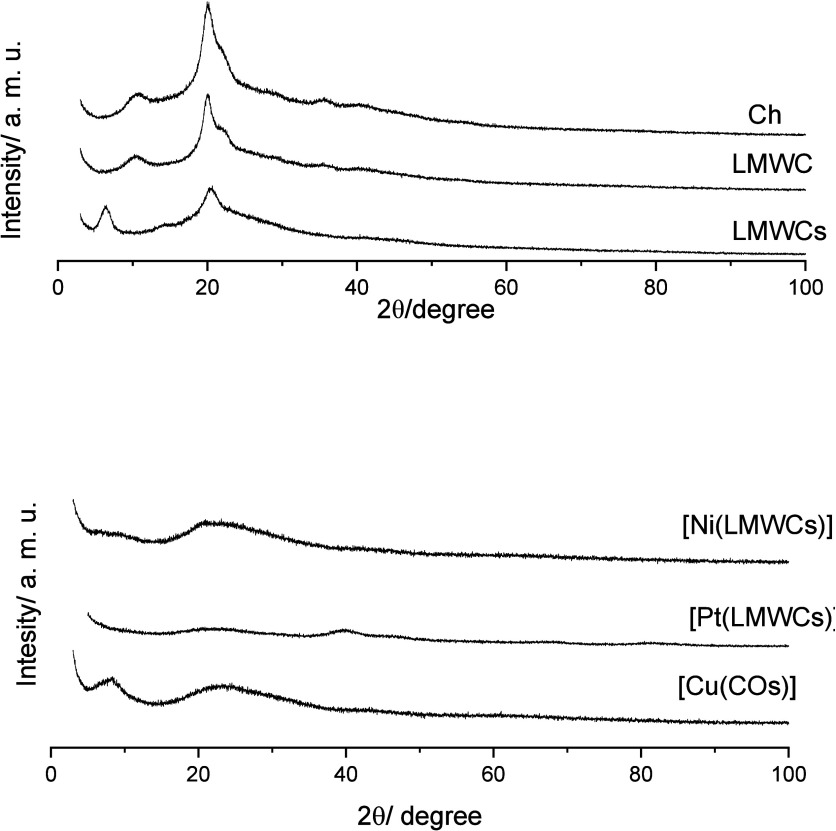
XRD diffraction patterns for Ch, LMWC, LMWC_s_, [Ni­(LMWC_s_)], [Pt­(LMWC_s_)], and [Cu­(LMWC_s_)].

### 3.6 Scanning Electron Microscopy (SEM)

The micrographs of Ch and LMWC (Figures 9a and 9b, respectively) revealed clusters with a rough surface and homogeneous-sized particles, consistent with a previous report.^37^ LMWC_s_ (Figure 9c) had a smooth surface and heterogeneous particle sizes. This change in the material’s morphology can be attributed to chemical modification and the formation of the Schiff base in NH_2_–C2 as well as to sample drying and grinding.^38^

**Figure 9:**
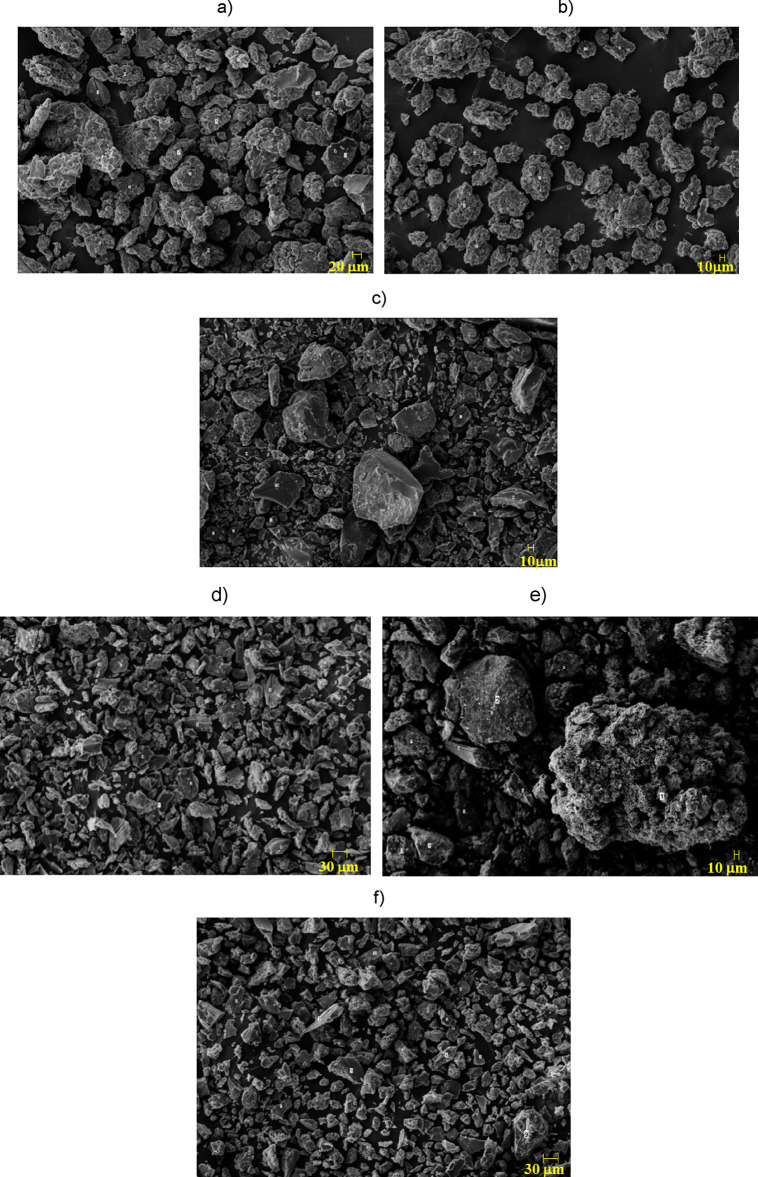
SEM images of unmodified chitosan particles Ch (a, b), LMWC (c), particles modified with salicylaldehyde (LMWC_s_), and respective complexes [M­(LMWC_s_)] (M= (d) Ni, (e) Pt, or (f) Cu).

The micrographs of the synthesized complexes [Ni­(LMWC_s_)], [Pt­(LMWC_s_)], and [Cu­(LMWC_s_)] presented in Figure 9d–f, respectively, demonstrate heterogeneity in particle size as well as both smooth and rough surfaces.

## 4 Catalytic Activity

The catalytic properties of chitosan (Ch), the free ligand (LMWC_s_), and derivative complexes [M^II^(LMWC_s_)] in the reaction between CO_2_ and styrene oxide for the synthesis of styrene carbonate were investigated. The resulting products from the carbonation reaction were characterized using FTIR and ^1^H NMR. The conversion of epoxide to carbonate was determined from ^1^H NMR spectra. Boroujeni et al.^16^ reported good results under optimized conditions: 3 mmol of styrene oxide, 0.03 mmol of TBAB as a cocatalyst, CO_2_ (1 atm), 0.025 g of the catalyst, and 4 h at less than 80 °C. Therefore, similar conditions were reproduced in the present study to determine catalytic activity, as shown in Figure 10 and in Table 5.

**Figure 10:**
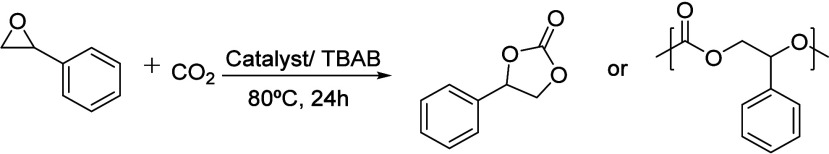
Synthesis of cyclic carbonate.

**Table 5:** Conversion of Styrene Oxide to Styrene Carbonate after 24 h at 80 °C Using 1 bar CO_2_

entry	**catalyst** ^a^	TBAB (mg)	conversion (%)
1	none	4.9	33.0
2	none	0.0	3.60
3	LMWC	4.9	74.2
4	LMWC_s_	4.9	65.4
5	[Cu(LMWC_s_)]	4.9	-^b^
6	[Ni(LMWC_s_)]	4.9	79.0
7	[Pt(LMWC_s_)]	4.9	82.5

a The catalyst loading was 15.0 mg.

b Polymerized.

Figure 11 shows the difference between the ^1^H NMR spectra of styrene oxide (SO) and the product resulting from the fixation reaction with CO_2_ using the LMWC and LMWC_s_ catalysts. Figure 11 also displays the spectra for the products formed using [Ni­(LMWC_s_)], [Cu­(LMWC_s_)], and [Pt­(LMWC_s_)] as catalysts. Three distinct signals were identified in the SO spectrum without a treatment. Hydrogen atoms attached to the carbons of the epoxide group exhibit chemical shifts at 2.80, 3.16, and 3.89 ppm.^2^ After the carbonation reaction, the signals attributable to SO either disappeared or decreased in intensity, indicating the formation of a cyclic carbonate. The spectra of the compounds formed after the CO_2_ fixation reaction exhibited new signals characteristic of cyclic carbonates in the region between 5.68 and 4.36 ppm, indicating that the reaction had occurred.^5,39^ These hydrogens exhibit higher chemical shifts as a result of increased shielding. For the product formed using [Cu­(LMWC_s_)] as the catalyst, signals were observed at 4.80, 3.72, and 3.64 ppm, consistent with the polymerization.

**Figure 11:**
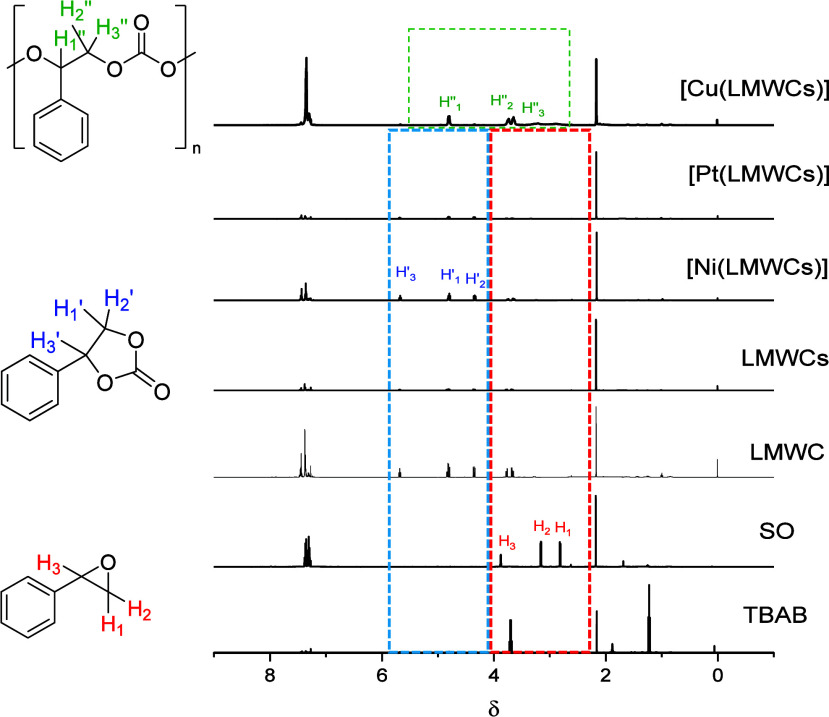
^1^H NMR spectra of styrene oxide and compounds formed after carbonation reaction using free ligands and metal complexes as catalysts and TBAB as a cocatalyst.

The results prove that all compounds prepared are active catalysts. Table 5 presents the conversion rates for the epoxide-to-carbonate conversion using different catalysts. Conversion rates were determined from the ^1^H NMR spectra.

In the absence of a catalyst, the reaction had a significant reduction in yield, and the conversion rate was 33% (Table 5, entry 1). The reaction in the presence of LMWC and LMWC_s_ provided a certain product yield (Table 5, entries 3 and 4). Among these catalysts, LMWC was the most effective, yielding 74% styrene carbonate.

The platinum catalyst exhibited good activity in the following reaction system: 1 bar CO_2_ at 80 °C, with a reaction time of 24 h. The aliquot analysis indicated 82.5% for the catalysis of [Pt­(LMWC_s_)] (Table 5, entry 7), whereas the analyses of the aliquots of the reactions using nickel indicated a conversion of 79.0% (Table 5, entry 6).

Although conversion to cyclic carbonates with the complexes was satisfactory under the above conditions, the metal center significantly influenced catalytic activity. Greater activity was observed for the palladium­(II) complex due to its higher reactivity, as nickel­(II) has a smaller ionic radius and platinum­(II) undergoes lanthanide contraction.^40^ Moreover, the ^1^H NMR spectrum suggests that the [Cu­(LMWC_s_)] complex polymerizes, leading to the formation of a polycarbonate. Therefore, this complex was not considered for the conversion calculation in Table 5.^41^

From a practical and sustainability perspective, the increase in conversion observed for Pt-LMWC may not necessarily justify the use of a precious metal catalyst in all applications. Nevertheless, the results are valuable for understanding the role of metal coordination in tuning the catalytic behavior of Schiff-base-modified low-molecular-weight chitosan systems.

A direct comparison with previously reported catalysts is not straightforward since the literature encompasses a broad variety of catalytic systems and experimental conditions, while closely related chitosan-derived Schiff base metal complexes remain scarcely explored. Therefore, the present study focused on assessing the feasibility and catalytic potential of the proposed materials by using a model CO_2_ cycloaddition system under standardized conditions.

Figure 12 shows the infrared spectra for the compounds formed from the CO_2_ fixation reaction with styrene oxide in the presence of the catalysts. The presence of bands at different wavenumbers compared to the SO bands is an indication of carbonate formation, specifically, the characteristic bands at 1803 cm^–1^ corresponding to the stretching of the carbonyl group *v*(C=O) and at 1052 cm^–1^ associated with the stretching of the −O-C = O–C bond in the five-membered ring. Furthermore, a band was found at 753 cm^–1^, corresponding to the C–H deformation characteristic of carbonates. The FTIR spectrum of the compound formed with [Cu­(LMWC_s_)] revealed a C=O band stretching close to 1785 cm^–1^. A band was also observed at approximately 1244 cm^–1^, corresponding to the C–O stretching vibration.^42^

**Figure 12:**
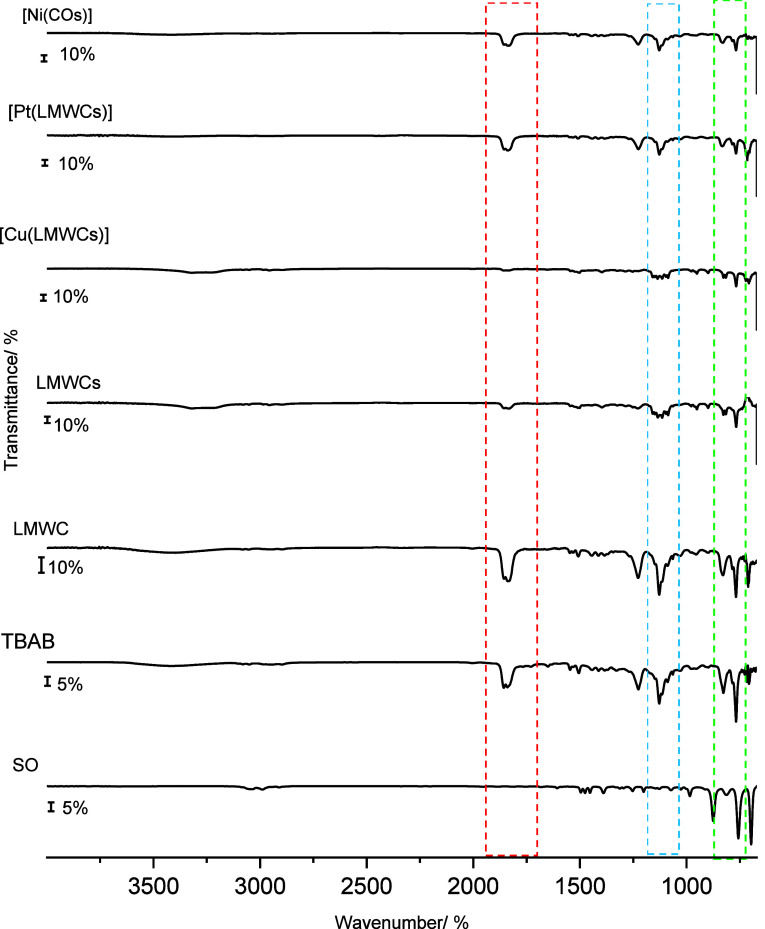
Absorption spectra in the infrared region for compounds obtained in carbonation reaction.

The synthesis of cyclic carbonates from CO_2_ and epoxides demonstrates a relationship among the −C=NC structure, imine group in the free LMWC_s_ ligand, and catalytic activity. However, the presence of an OH substituent group in the aromatic ring induces resonance in the system. Therefore, hydroxyl acts as a Lewis base and plays an important role in the activation of the CO_2_.

First, the hydrogen atom of the hydroxyl group coordinates with the oxygen of the epoxide via hydrogen bonding, thereby activating the epoxide. Simultaneously, a nucleophilic attack of the bromide ion (Br^–^) from the cocatalyst (TBAB) occurs on the less sterically hindered carbon atom, leading to the opening of the epoxide ring (step I). The negatively charged oxygen atom of the alkoxide intermediate reacts with CO_2_ to form an intermediate (step II). In the subsequent step, CO_2_ is inserted into the C–O bond of the epoxide to form intermediate III. Lastly, the cyclic carbonate is formed by ring closure through an intramolecular nucleophilic attack and the catalyst is regenerated. A proposed mechanism, based on studies in the literature, is shown in Figure 13.^3,43^

**Figure 13:**
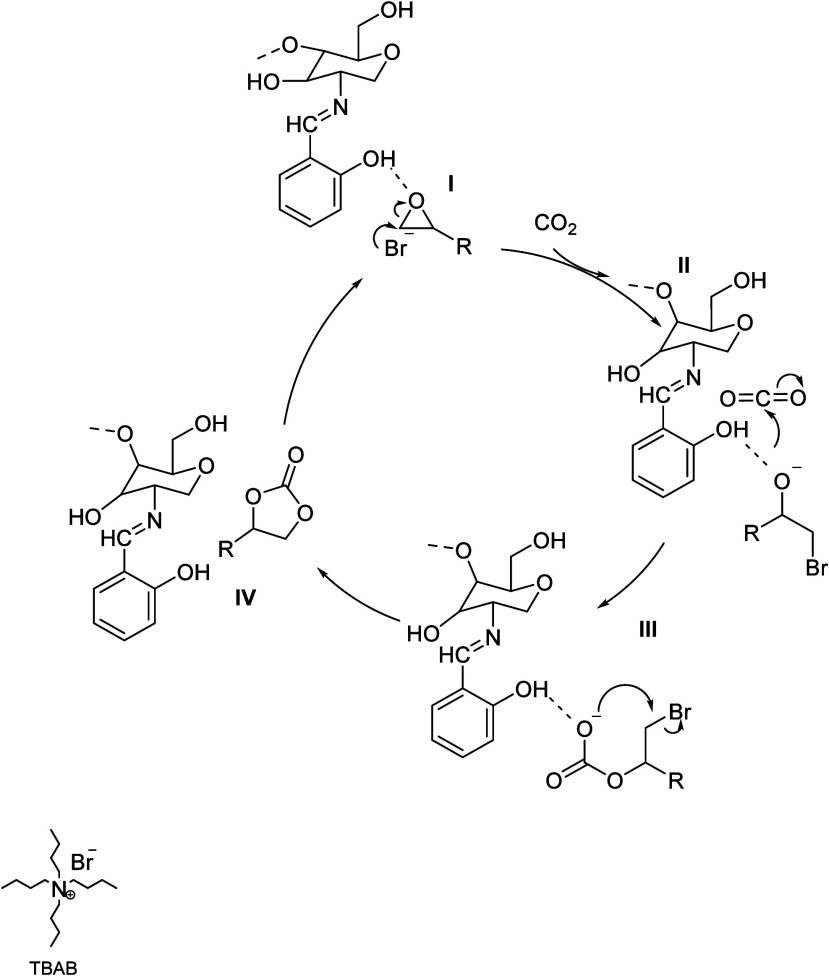
Proposed reaction mechanism.

The metal complex behaves as a Lewis acid. The metal complex coordinates with the epoxide, thereby activating ring-opening. The nucleophilic bromide ion from the TBAB cocatalyst attacks the epoxide, preferably at the less hindered face, where the epoxide is bonded to the metal. This attack opens the epoxide ring, forming an alkoxide intermediate bound to the metal via an S_N_2 (bimolecular nucleophilic substitution) reaction. Next, CO_2_ is added to the alkoxide intermediate, thereby forming a metal-bound carbonate. This metal carbonate subsequently undergoes a cyclization reaction (S_N_2), thus regenerating the catalyst.^43^ A proposed mechanism, based on studies in the literature, is shown in Figure 14.^16,44^

**Figure 14:**
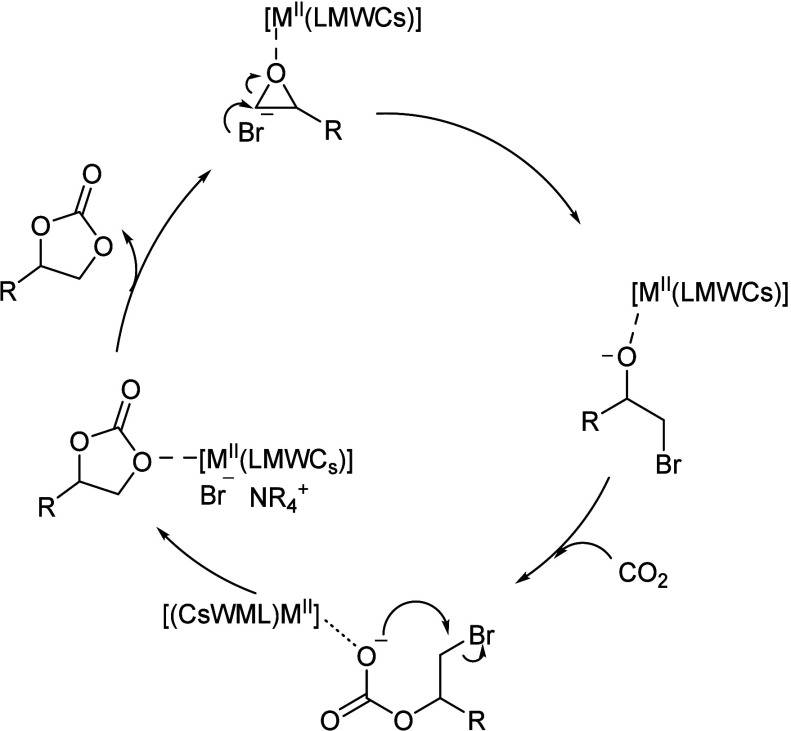
Proposed potential reaction mechanism.

Regarding the differences in efficiency observed in Table 5, a possible explanation for the lower catalytic efficiency of the Schiff base formed with low-molecular-weight chitosan (LMWCs) compared to unmodified chitosan is that the −NH_2_ groups present in LMWC are more nucleophilic and basic than the −OH groups in the Schiff base. The amino groups in LMWC are more readily available for interaction with reaction intermediates, potentially facilitating the catalytic process more efficiently than the less nucleophilic hydroxyl groups in the Schiff base. This difference in nucleophilicity and basicity could explain the reduced catalytic activity of the Schiff base in the CO_2_ cycloaddition reaction.

Regarding the behavior of the metal complexes, the platinum one typically exhibits superior catalytic activity in CO_2_ cycloaddition reactions due to its ability to exist in multiple stable oxidation states. The possibility of platinum­(II) oxidation makes it a better Lewis acid and seems to play an important role in stabilizing reaction intermediates through electronic interactions and coordination, thereby facilitating the desired reaction pathway. In contrast, nickel is less effective because its redox chemistry is more limited, primarily relying on Ni­(II) and Ni(0) states, which are less able to stabilize intermediates and can result in a lower catalytic efficiency.

No articles were found reporting Cu (II) complexes in the styrene oxide/CO_2_ polymerization, but there were articles reporting Co, Cr, Zn, and Al complexes. Based on the articles and the literature findings, it is possible to suppose that the preferential formation of polymeric products over cyclic carbonates may be attributed to the Lewis acidic characteristics and the coordination environment of the Cu­(II) center. Previous mechanistic investigations into CO_2_/epoxide copolymerization have demonstrated that the metal center is fundamentally involved in activating the epoxide and stabilizing propagating alkoxide intermediates. Specifically, metal-mediated polarization of the epoxide C–O bond facilitates ring-opening and the formation of metal-alkoxide species, which then undergo CO_2_ insertion and chain propagation rather than intramolecular cyclization. The mechanistic behavior has been extensively reported for other metal catalysts, predominantly complexes of Co, Cr, Zn, and Al, which are among the most extensively studied systems for CO_2_/epoxide ROCOP.^45–47^

Furthermore, the selectivity between cyclic carbonate formation and polymer propagation is highly dependent on the catalyst structure, coordination environment, and stabilization of growing carbonate/alkoxide species. Catalysts capable of maintaining propagating intermediates coordinated to the metal center may suppress backbiting reactions responsible for cyclic carbonate formation.^45–47^

In the present case, the Cu-LMWC system likely favors the stabilization of propagating Cu-alkoxide/carbonate intermediates through Lewis acidic activation of styrene oxide, thereby promoting chain-growth pathways over cyclic carbonate formation. Although the relatively small ionic radius and high charge density of Cu­(II) may contribute to stronger epoxide polarization, the observed selectivity is more plausibly associated with the coordination-mediated stabilization of propagating species rather than with redox cycling processes for which no direct experimental evidence was observed under the current experimental conditions.

However, further studies would be required to confirm these hypotheses, as a more detailed understanding of the reaction mechanisms and metal behavior in various oxidation states is necessary. These aspects are beyond the scope of this work.

## Conclusions

Low-molecular-weight chitosan-derived Schiff base complexes containing Ni­(II), Pt­(II), and Cu­(II) were successfully synthesized through a simple and sustainable route. Spectroscopic analyses by FTIR and NMR confirmed the formation of the Schiff base and its coordination with the metallic centers, while Raman spectroscopy provided additional evidence of Cu­(II) coordination. Structural and physicochemical characterization demonstrated that the chemical modification and complexation processes significantly altered the properties of the original chitosan matrix. XRD analyses revealed a substantial reduction in crystallinity after Schiff base formation and metal coordination, whereas SEM micrographs showed pronounced morphological changes in the modified materials. Thermal analyses further demonstrated that metal coordination increased the thermal stability of the biopolymeric systems, particularly for the Pt­(II)-containing complexes.

The synthesized materials were successfully applied as catalytic systems for the cycloaddition of CO_2_ to styrene oxide under mild conditions (80 °C and 1 bar CO_2_). All catalytic systems exhibited higher activity than the noncatalyzed reaction, confirming the catalytic potential of the chitosan-derived materials. Among the investigated complexes, the Pt­(II) and Ni­(II) systems showed the highest conversions to styrene carbonate, whereas the Cu­(II) complex exhibited a tendency toward polymerization. These results indicate that both the Schiff base environment and the nature of the coordinated metal center strongly influence catalytic behavior.

Overall, this work demonstrates that chitosan-derived Schiff base metal complexes are promising and sustainable catalytic platforms for CO_2_ valorization. In addition to providing efficient catalytic activity under relatively mild conditions, the materials combine renewable biopolymeric matrices with tunable structural and physicochemical properties, highlighting their potential for future development in sustainable catalytic systems.

## Supplementary Material



## Data Availability

Authors can confirm that all relevant data are available throughout the manuscript and supporting file.

## Notes

^13^C NMR spectra of LMWC and LMWC_s_; FTIR spectra of [Ni­(LMWC_s_)], [Pt­(LMWC_s_)], and [Cu­(LMWC_s_)]; and Raman spectra of copper II complexes with LMWC_s_ (PDF)
