# Supplementary material for: Schiff-Base-Modified Chitosan-Metal Complexes as Catalysts in CO2 Cycloaddition Reactions
Source: ACS Omega. 2026 Jun 30;11(27):39990–40004. doi: 10.1021/acsomega.6c01736 (PMC13382743; doi:10.1021/acsomega.6c01736)

Schiff-Base-Modified Chitosan-Metal Complexes as Catalysts in CO_2 Cycloaddition Reactions

Jackelinne Camargo de Lima[a], Rafael Turra Alarcon[a], Gilbert Bannach[b], Ana Paula Garcia Ferreira[a], Carla Cristina Schmitt[a], Éder Tadeu Gomes Cavalheiro[a*]

[a] Instituto de Química de São Carlos, Universidade de São Paulo,
Av. Trabalhador São Carlense, 400, 13566-590, São Carlos, SP, Brazil

[b] Faculdade de Ciências, Universidade Estadual Paulista Campus Bauru,
Av. Eng. Luiz Edmundo Carrijo Coube, 14-01, 17033-360, Bauru, SP, Brazil

E-mail: *cavalheiro@usp.br

Supplementary material

^{13}C NMR

Figure S1 ^{13}C NMR spectra LMWC and LMWCs

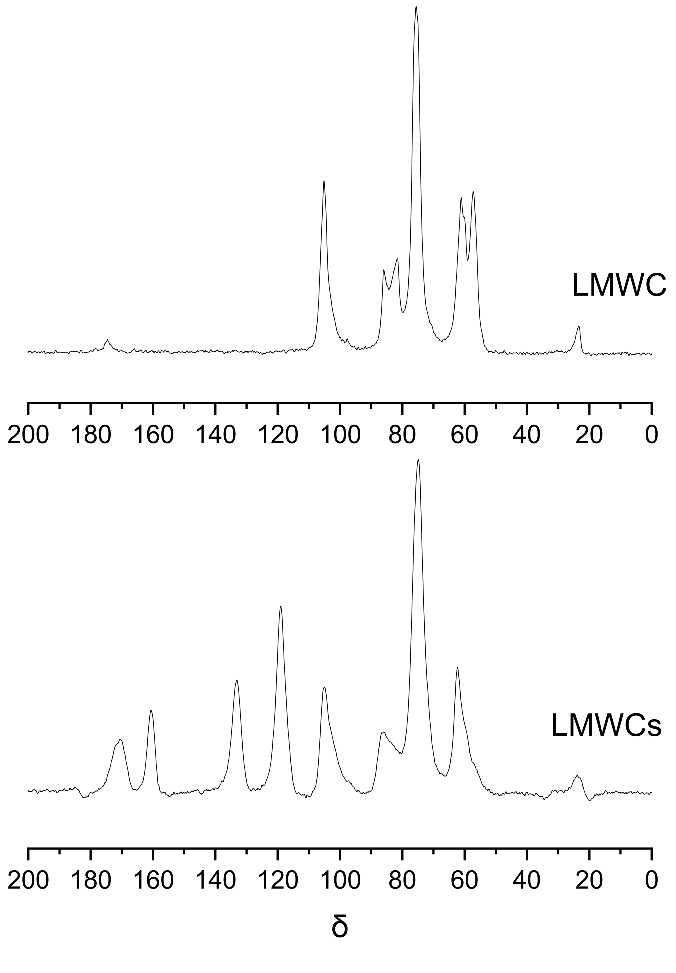

Infrared spectra (FTIR)

Figure S2

(a) Infrared spectrum of depolymerized chitosan complex with salicylaldehyde and Ni(II) ATR.

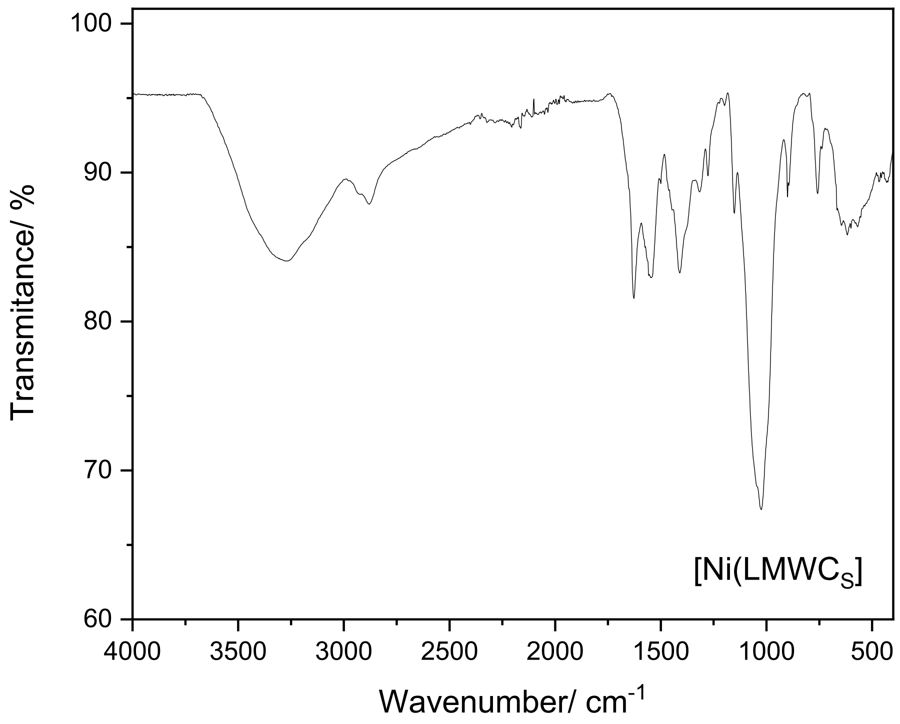

(b) Infrared spectrum depolymerized chitosan complex with salicylaldehyde and Ni(II) CsI.

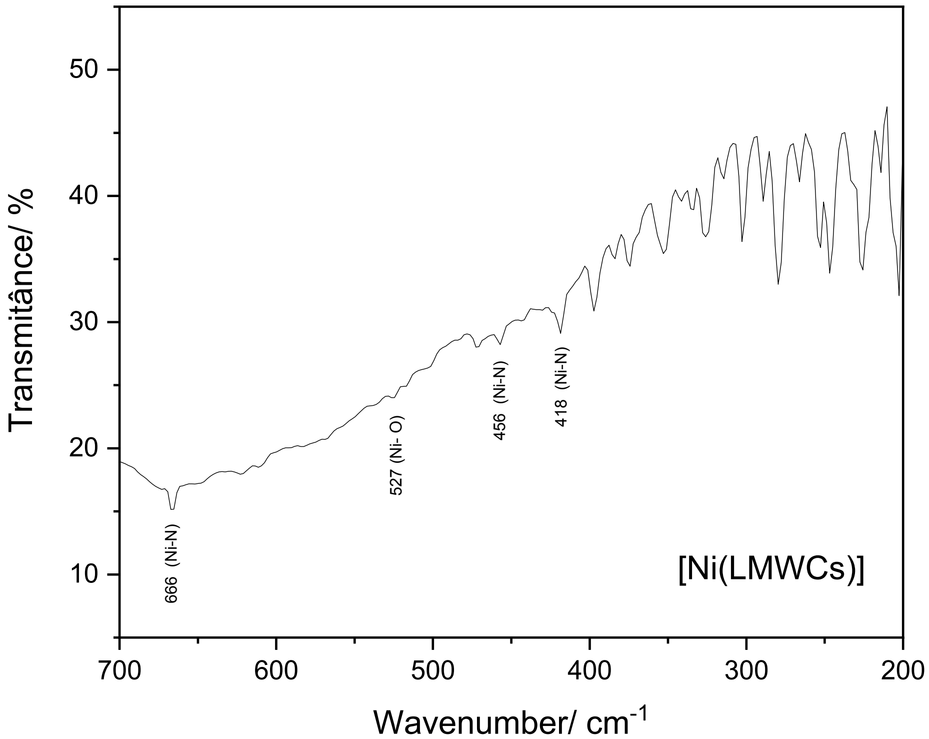

Figure S3 - Infrared spectrum depolymerized chitosan complex with salicylaldehyde and Pt(II) ATR.

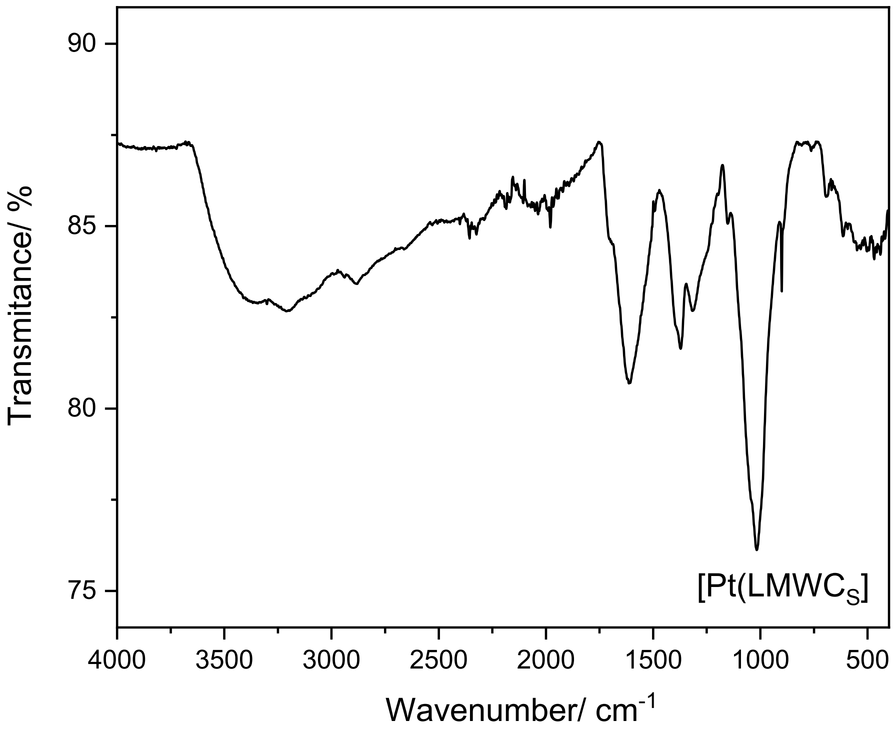

Figure S4

(a) Infrared spectrum depolymerized chitosan complex with salicylaldehyde and Cu(II) ATR.

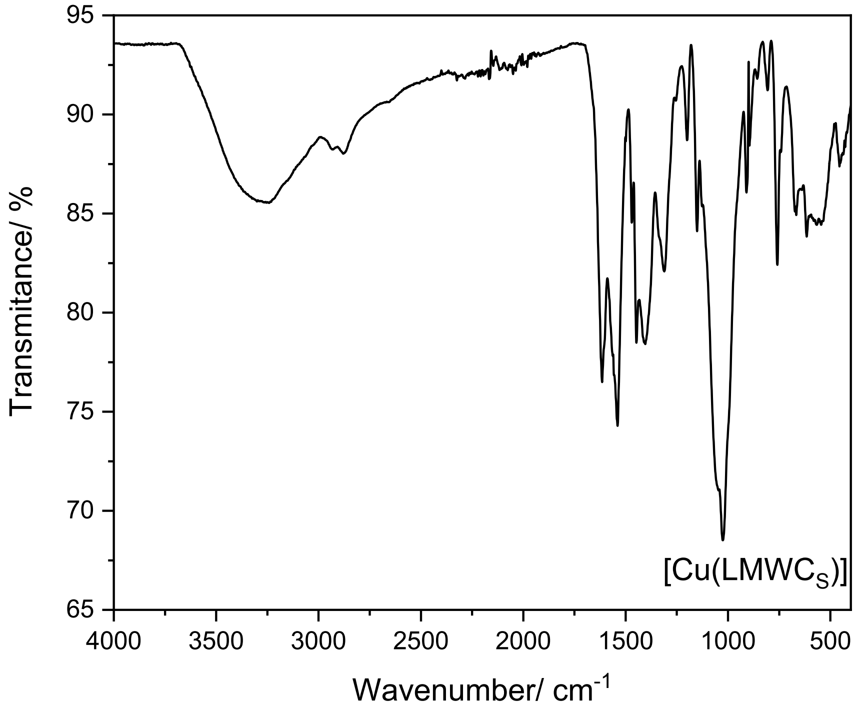

(b) Infrared spectrum depolymerized chitosan complex with salicylaldehyde and Cu(II) CsI.

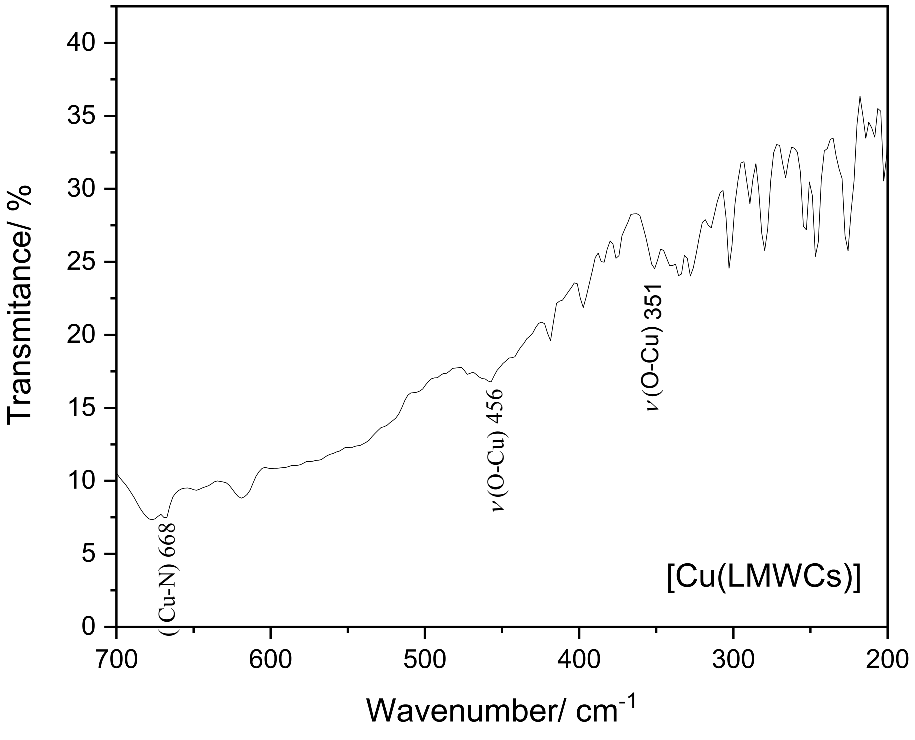

Raman spectroscopy

Figure S5- Raman spectrum of the [Cu(LMWCs)] complex.

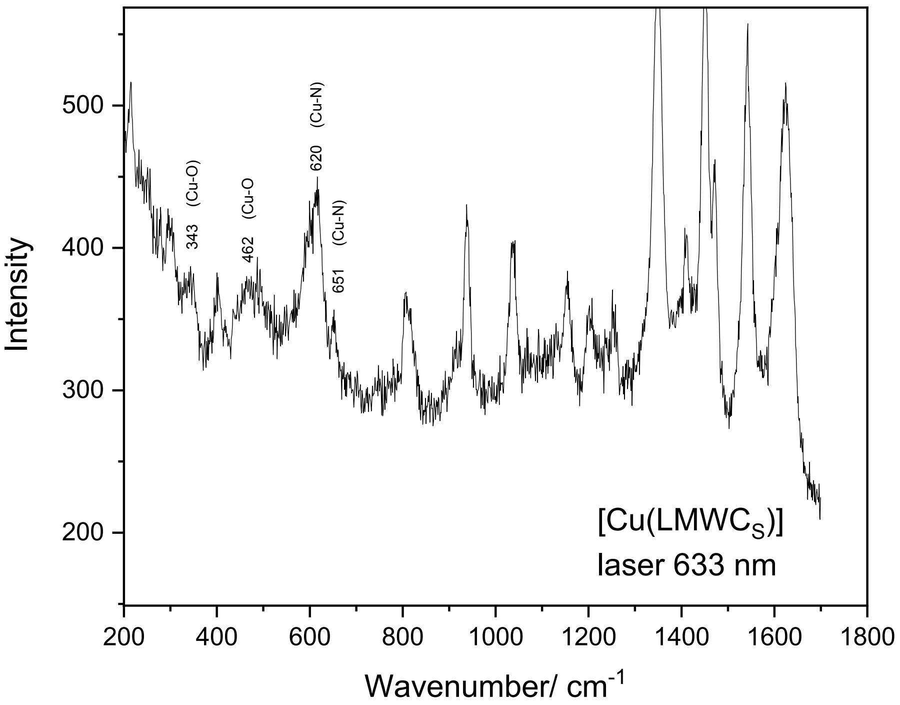

Supplement: Supplementary file 1 [file ao6c01736_si_001.pdf]
